# Optimization and Lead Selection of Benzothiazole Amide Analogs Toward a Novel Antimycobacterial Agent

**DOI:** 10.3389/fmicb.2018.02231

**Published:** 2018-09-20

**Authors:** Mary A. De Groote, Thale C. Jarvis, Christina Wong, James Graham, Teresa Hoang, Casey L. Young, Wendy Ribble, Joshua Day, Wei Li, Mary Jackson, Mercedes Gonzalez-Juarrero, Xicheng Sun, Urs A. Ochsner

**Affiliations:** ^1^Mycobacteria Research Laboratories, Department of Microbiology, Immunology and Pathology, Colorado State University, Fort Collins, CO, United States; ^2^Crestone, Inc., Boulder, CO, United States

**Keywords:** NTM, tuberculosis, antibacterial therapeutics, benzothiazole amide, MmpL3, efficacy, tolerability, aerosol

## Abstract

Mycobacteria remain an important problem worldwide, especially drug resistant human pathogens. Novel therapeutics are urgently needed to tackle both drug-resistant tuberculosis (TB) and difficult-to-treat infections with nontuberculous mycobacteria (NTM). Benzothiazole adamantyl amide had previously emerged as a high throughput screening hit against *M. tuberculosis* (*Mtb*) and was subsequently found to be active against NTM as well. For lead optimization, we applied an iterative process of design, synthesis and screening of several 100 analogs to improve antibacterial potency as well as physicochemical and pharmacological properties to ultimately achieve efficacy. Replacement of the adamantyl group with cyclohexyl derivatives, including bicyclic moieties, resulted in advanced lead compounds that showed excellent potency and a mycobacteria-specific spectrum of activity. MIC values ranged from 0.03 to 0.12 μg/mL against *M. abscessus* (*Mabs*) and other rapid- growing NTM, 1–2 μg/mL against *M. avium* complex (MAC), and 0.12–0.5 μg/mL against *Mtb*. No pre-existing resistance was found in a collection of *n* = 54 clinical isolates of rapid-growing NTM. Unlike many antibacterial agents commonly used to treat mycobacterial infections, benzothiazole amides demonstrated bactericidal effects against both *Mtb* and *Mabs*. Metabolic labeling provided evidence that the compounds affect the transfer of mycolic acids to their cell envelope acceptors in mycobacteria. Mapping of resistance mutations pointed to the trehalose monomycolate transporter (MmpL3) as the most likely target. *In vivo* efficacy and tolerability of a benzothiazole amide was demonstrated in a mouse model of chronic NTM lung infection with *Mabs*. Once daily dosing over 4 weeks by intrapulmonary microspray administration as 5% corn oil/saline emulsion achieved statistically significant CFU reductions compared to vehicle control and non-inferiority compared to azithromycin. The benzothiazole amides hold promise for development of a novel therapeutic agent with broad antimycobacterial activity, though further work is needed to develop drug formulations for direct intrapulmonary delivery via aerosol.

## Introduction

Infections with non-tuberculous mycobacteria (NTM) are increasing in incidence (Falkinham, 2013; Prevots and Marras, 2015; Strollo et al., 2015; Vinnard et al., 2016; Prevots et al., 2017; Spaulding et al., 2017) and are notoriously difficult to treat (Henkle et al., 2017; Lande et al., 2018). Resistance of NTM to disinfectants may contribute to hospital acquired infections (Caskey et al., 2018) especially in cystic fibrosis disease where infection is common as patients age (Nick et al., 2016; Martiniano et al., 2017). Increasing evidence suggests that there is a protective effectiveness of the Bacillus Calmette-Guerin (BCG) vaccination against NTM disease (Zimmermann et al., 2018). Tuberculosis may give some cross-protection to NTM infection. With the declining incidence of TB in the US, NTM may fill this immunity void and we will see rising numbers of cases and will be challenged with treating NTM infection as the tuberculosis prevalence is at an all-time low in the US. Given that the US has never undertaken a BCG vaccination strategy like most other nations there may be a niche for NTM infections. NTM are ubiquitous in the environment and disease due to these organisms are heterogeneous disorders with pulmonary manifestations being the most common presentation. Lung disease manifests as nodular bronchiectasis and/or fibrocavitary disease. Some individuals will remain culture positive but clinically stable, but those with significant respiratory symptoms and radiographic abnormalities including destructive cavitary manifestations and microbiological evidence of an NTM will require treatment as disease progression frequently occurs and mortality can be high (Fleshner et al., 2016). Increasing prevalence of NTM will continue to occur as the population ages (Falkinham, 2003). Immune dysfunction has been seen in patients with pulmonary NTM (Cowman et al., 2018). Since many patients have underlying host predispositions they are susceptible to re-infection and may need intermittent treatment when symptomatic infection recurs (Lake et al., 2016). Treatment of non-tuberculous mycobacterial lung disease (NTM-LD) is challenging for several reasons including the relative resistance of NTM to currently available drugs and the difficulty in tolerating prolonged treatment with multiple drugs (Philley et al., 2016).

There are virtually no antimicrobial drug discovery programs specifically targeting NTM. Although therapeutic agents developed to treat *Mtb* infections often lack activity against NTM, it remains an attractive approach to initiate new NTM drug discovery projects via screening a library of TB active compounds against NTM, which has indeed resulted in high hit rates (Low et al., 2017). In fact, our development candidate, benzothiazole adamantyl amide, was discovered via that approach in a screening for *Mabs* whole cell activity of a library composed of hits from a previous *Mtb* screening (Franzblau et al., 2012). Another strategy for NTM drug discovery is to revisit or repurpose older drugs. A potential role for clofazimine in NTM treatment regimens has been suggested, since this agent showed bactericidal activity and synergy with amikacin or clarithromycin, both of which are commonly used antibiotics to treat NTM infections (Ferro et al., 2016). Semisynthetic spectinamides derived from the old spectinomycin discovered over half a century ago have shown potent activity against MDR and XDR tuberculosis (Liu et al., 2017).

NTM treatment regimens differ by species (Daley and Glassroth, 2014; Kasperbauer and De Groote, 2015), particularly between rapid growers (RGM) comprised of *M. abscessus* (*Mabs*) complex (*M. abscessus*, ssp. *abscessus, bolletti*, and *massiliensis*), *M. chelonae, M. fortuitum*, and others; and slow growers represented by *M. avium, M. intracellulare*, and *M. chimaera* (*M. avium* complex, or MAC). *M. abscessus* (*Mabs*) is particularly difficult to treat (Haworth et al., 2017). This organism is capable of forming more virulent rough phenotypes and smooth forms that tend to form biofilms (Claeys and Robinson, 2018) associated with antibiotic drug resistance (Clary et al., 2018). Resistance to macrolides, a cornerstone class of therapeutic agents, is innate in certain *Mabs* subspecies that carry an inducible methylase genetic element and the presence of this gene affects the outcomes (Koh, 2017; Choi et al., 2018). This resistance is detected clinically as well as in the laboratory (Carvalho et al., 2018) and is an important development limiting effective therapy (Kasperbauer and De Groote, 2015). Therapy involves multiple agents and recalcitrant or severe infections are optimally managed with the addition of an injectable agent (De Groote and Huitt, 2006; Philley et al., 2016). Therapeutic intolerances and side effects limit adherence to treatment regimens and affect outcomes. In addition, the potency of available agents is relatively low. Thus, there is a compelling need for novel antibiotics that are more potent and better tolerated.

The most common form of NTM disease is lung disease. NTM can result in chronic progressive lung disease especially for those with known risk factors, the most common of which is aging. Patients with cystic fibrosis, chest structural abnormalities and pre-existing lung disease such as bronchiectasis and autoimmune diseases and their treatments are all risk factors for infection and disease. For pulmonary disease, direct delivery to the airway would allow greater penetration and less systemic exposure. An inhaled route of delivery would be advantageous for intermittent therapy, in particular to suppress systemic side effects. Advances have been made in the area of aerosol delivery carriers and devices. The success of inhalational therapies with liposomal amikacin (Olivier et al., 2014; Caimmi et al., 2018) and Arikayce™ (Olivier et al., 2017) has paved the way for new therapies in this category. The development of a multicenter clinical trial network will allow more rapid enrollment in new drug treatment trials (Kevin Winthrop, personal communication).

Much remains to be done in the field of NTM as this has been a neglected area of drug development. As more treatment response surrogate markers are discovered, the future holds great promise for utilization of biomarkers to match therapeutic regimens to appropriate patient subpopulations and to monitor treatment response (Asakura et al., 2015) similar to biomarker work in TB to enhance drug development (Sigal et al., 2017). This will require clinical studies involving multi-center cooperative studies to ensure an adequate number of enrollees. In addition, diseases like Buruli ulcer caused by *M. ulcerans* are emerging infections in need of novel treatment approaches (Tai et al., 2018; Zingue et al., 2018). Wound healing is prolonged and problematic (O'Brien et al., 2018) and topical therapy for Buruli ulcer or *M. marinum* cutaneous infections may have some utility depending on drug penetration into deeper tissues (Simoes et al., 2016). Mycobacteria live in lipid-rich host environments (Aguilar-Ayala et al., 2018; Ayyappan et al., 2018) so a lipophilic drug might be attractive for a variety of disease manifestations if lipophilicity can be managed. There is always the need to balance good drug properties such as hydrophilicity with the impenetrable lipid rich cell walls of mycobacteria. In addition to delivery improvements, future work to optimize potency against members of the slowly growing mycobacteria, particularly (MAC), will be a priority.

## Materials and methods

### Synthesis of benzothiazole amide analogs

Unsubstituted benzothiazole adamantyl amide had emerged as a hit during screening of a focused library of TB-actives (Franzblau et al., 2012) for compounds that also possessed activity against *M. abscessus*. This scaffold represented the starting point for a campaign to elucidate the structure-activity relationship in greater detail. The initial strategy involved trimming of the adamantyl group to a minimum of lipophilic structure required for activity. The general synthetic route started from substituted 2-amino-benzothiazole intermediates and variably substituted cycloalkyl carboxylic acids under standard amide coupling conditions using 1-[Bis(dimethylamino)methylene]-1H-1,2,3-triazolo[4,5-b]pyridinium 3-oxide hexafluoro-phosphate (HATU) in the presence of N,N-diisopropylethylamine (DIEA) in dichloroethane (DCE). A detailed description of chemical synthesis, purification and SAR of substituted benzothiazole cyclohexyl amides is presented elsewhere (Graham et al., 2018). The products were purified by column chromatography using ethyl acetate and hexanes as eluents and fully characterized by NMR and LC-MS. Purity of the lead analogs described here was >95%.

### Microbiological evaluation of benzothiazole amide compounds

NTM clinical isolates were from University of Colorado Hospital (De Groote et al., 2014a). *M. chimaera* strains were provided by the CDC (van Ingen et al., 2017), Reference strain *Mabs* 19977 and other bacterial strains, including anaerobes, were from ATCC, BEI Resources, or from the biorepository at Crestone, Inc. The avirulent *Mtb* strain H37Rv mc^2^ 6206 is a *leuC leuD* mutant derivative of strain mc^2^ 6020 (Δ*lysA* Δ*panCD*) and was provided by Dr. Bill Jacobs (Sambandamurthy et al., 2005). Compounds were tested for antimicrobial activity against mycobacteria and other pathogens following guidelines published by the Clinical Laboratory Standards Institute (CLSI). Muller-Hinton broth (MHB, 3 days) was used for rapid-growing NTM and Middlebrook medium (7H9 + OADC, 7 days) for slow-growing NTM and for *Mtb* (CLSI, 2003). Accurate endpoint minimum inhibitory concentration (MIC) values were obtained after addition of resazurin for 24 h to monitor viable cells colorimetrically (Khalifa et al., 2013). Protein and serum binding of compounds was tested by MIC determination in the presence of human albumin (45 mg/mL) or 50% complement-inactivated human serum. Potential non-specific effects on membranes were monitored in a hemolysis assay, where equine erythrocytes were exposed to compounds over a concentration range of up to 128 μg/mL in 10 mM Tris-HCl buffered 0.9% saline (pH 7.5) for 10 min at room temperature, centrifuged and evaluated for absence of erythrocyte pellets and buffer discoloration due to hemolysis. Spontaneous resistant mutants were isolated by plating 10^9^-10^10^ *Mabs* ATCC 19977 cells onto 7H11-ADC agar plates containing benzothiazole amide compounds at multiples of their MIC, followed by colony purification on selective agar, preparation of genomic DNA, PCR amplification and sequencing of the *mmpL3* gene. For time-kill assays, cultures of *Mabs* and *Mtb* were diluted with broth to cell densities of 10^5^-10^6^ CFU/mL, compounds and control agents were added at 10 times their MICs and samples were removed for colony enumeration over a period of 5 days (*Mabs*) and 21 days (*Mtb*). Synergy studies applied the MIC checkerboard method of CRS400393 with 10 commercially available antibiotics, and the fractional inhibitory concentration index (FIC-I) was determined (Li et al., 2017). Metabolic labeling assays using [^14^C]acetate of *Mabs* ATCC19977 cultures either untreated or treated with benzothiazole amides at 2 and 10 times their MICs and lipid extraction and analysis were performed as described (Li et al., 2014).

### Preliminary determination of pharmacological properties

ADMET assays were performed through NIAID Preclinical Services and Eurofins. Potential for cytotoxicity was monitored in HepG2 cells after 72 h growth in the presence of test compounds, either prepared as 10-point 3-fold serial dilutions or at a fixed 10 μM concentration. Cell viability was measured using the CellTiter-Glo® Luminescent Cell Viability Assay (Promega) or via fluorescent image analysis. Cytotoxicity was expressed as IC_50_ or as percent reduction of viable cells relative to a reference compound. Metabolic stability of compounds was evaluated via incubation with human liver microsomes at 37°C and quantitative analysis of parent compound by LC-MS/MS in samples removed at 0, 5, 15, 30, and 45 min (Obach, 1999). For cytochrome P450 (CYP) inhibition assays, compounds were prepared as a 7-point dilution series and incubated with human liver microsomes in buffer containing 2 mM NADPH and probe substrate. After incubation at 37°C for the optimal time (10–60 min) the reactions were processed for quantitative LC-MS/MS analysis of probe substrate metabolites in order to calculate IC_50_ values (Walsky and Obach, 2004). Thermodynamic solubility of compounds in aqueous media was determined by incubation in 50 mM potassium phosphate buffer (pH 7.4) for 24 h, followed by HPLC-UV detection of compound in the filtrate (Analiza, Cleveland, OH, United States).

### Efficacy and tolerability assessment

*In vivo* assessment of efficacy and tolerability of benzothiazole amide CRS400226 was assessed in a granulocyte macrophage colony stimulated factor-knockout (GM-CSF KO) mouse model of *Mabs* infection (Gonzalez-Juarrero et al., 2012; De Groote et al., 2014b). This study was carried out at Colorado State University which maintains a centralized IACUC registered by the USDA and accredited by the Association for Assessment and Accreditation of Laboratory Animal Care (AAALAC) International. The protocols were reviewed and approved by the CSU Animal Care and Usage Committee (ACUC) prior to development of the experimental infections. Male GM/CSF-KO mice were bred from the Gonzalez-Juarrero Lab colony at Colorado State University in the Painter Center for Laboratory Animal Resources. Mice at least 2 months old and at least 20 grams of weight were selected and acclimated for 1 week in the BSL3 laboratory prior to infection. Inoculum was prepared from a stock vial of *Mabs* strain #21, a clinical isolate, in 4 mL ddH2O to a targeted dose of 10^6^ CFU/50 μL. The actual CFU in the inoculum used for infection was determined by plating of serial dilutions onto 7H11 agar plates, followed by colony enumeration after 4 days. The mice were infected via intratracheal aerosol delivery using a Penn-Century microsprayer. Actual bacterial deposition in the lungs was determined in three mice that were sacrificed to remove the lungs for bead homogenization in 500 uL of PBS, and CFU determination in serial dilutions of the homogenate. Established infections were confirmed after 10 days, again by determination of CFU in the lungs of three mice. Post infection, animals were monitored daily and weights recorded weekly, to ensure <20% weight loss and to adjust drug dosing to the actual weight. Four groups of *n* = 5 mice were included in the study, and treatment started 10 days post infection. The benzothiazole amide CRS400226 was formulated in saline containing 5% corn oil and 0.05% Tween-80. CRS400226 and vehicle were administered via intrapulmonary liquid aerosol delivery (50 μL/dose) via liquid Penn-Century MicroSprayer (liquid) 5 days per week over a 4-week treatment course. Azithromycin (TOCRIS #1A/203325) was prepared as a solution in 0.6% acetic acid and was administered via oral gavage of 0.1 mL to achieve a dose of 100 mg/kg. One group of mice served as untreated controls. After 4 weeks of treatment, the mice were sacrificed and the lung lobes separated. The left lobes of the lungs were homogenized in PBS and plated to determine bacterial load. The middle right lobes were placed in 4% paraformaldehyde and processed for histology.

## Results

### Improved potency of advanced lead compounds and *in vitro* profiling

Following previous screening of compound collections against *Mtb* (Falzari et al., 2005; Franzblau et al., 2012), initial hits with modest activity against *Mabs* ATCC 19977 emerged from a whole cell activity screen of small molecule compound libraries. This led to the identification of scaffolds with broad antimycobacterial spectrum, such as the adamantyl amides. We applied an iterative process of design-synthesis-screen and increased potency by 1–2 orders of magnitude compared to the initial library hits (Graham et al., 2018). Optimization of the adamantyl amide series resulted in compound CRS400226, an adamantyl benzothiazole amide. Due to its high lipophilicity and consequent potential for nonspecific binding, the adamantyl group was eventually replaced with cyclohexyl derivatives, such as in CRS400153. Some loss of activity, particularly against slow-growing NTM, was noted when comparing the cyclohexyl derivatives to adamantyl compounds. A breakthrough in structure-activity relationship was achieved with bicyclic moieties, such as in CRS400359 and CRS400393, which resulted in improved potency against both rapid- and slow-growing NTM as well against *Mtb*. The properties of these advanced leads with regard to *in vitro* activity and ADMET profiles are shown in Table 1. The lead compounds demonstrated a very narrow, mycobacteria-specific spectrum of activity, without appreciable activity against other aerobic or anaerobic bacteria. *In vitro* activity and MIC distributions were further investigated for the top compounds against rapid-growing NTM clinical isolates. MIC ranges and MIC_90_ values, defined as the minimum concentration required to inhibit growth of at least 90% of all strains tested, are shown in Table 2. Advanced lead compounds had MIC_90_ of ≤ 1 μg/mL against all mycobacteria tested, which included *n* = 20 *Mabs, n* = 11 *M. chelonae, n* = 11 *M. fortuitum*, and *n* = 12 other rapid-growing NTM. Outlier strains with elevated MIC values for benzothiazole amides were not found and the MIC ranges were tight, while commonly used antibiotics such as amikacin, linezolid, and azithromycin showed bimodal MIC distributions (Supplementary Figure S1). Reduced susceptibility (MIC ≥ 8 μg/mL) to at least one of these three antibiotics was observed for 45 of 54 (83%) of the clinical isolates. The benzothiazole amides were equally active against drug-resistant strains, which is consistent with the absence of preexisting resistance to an agent directed against a novel target. Lead compound CRS400393 demonstrated MIC values ranging from ≤ 0.03 to 0.5 μg/mL against rapid-growing NTM, MIC = 1–2 μg/mL against MAC, and MIC ≤ 0.12 μg/mL against *Mtb*. Notably, all mycobacterial species were susceptible, including isoniazid, rifampin, or fluoroquinolone resistant strains of *Mtb*, and activity was maintained under low oxygen conditions and against intracellular *Mtb*, as shown for CRS400226 (Supplementary Table S2). In addition, lead compounds have so far shown favorable *in vitro* ADMET properties with regard to cytotoxicity, hemolysis, CYP inhibition, and metabolic stability, although *in vivo* safety and toxicity data have not yet been generated for the more potent analogs. Aqueous solubility was low (<3 μM) and protein binding was high (>95%) for all compounds of this series, likely attributable to the highly hydrophobic moieties of the compounds.

**Table 1:** *In vitro* activity of advanced benzothiazole amides.

**Compound**	**CRS400226**	**CRS400153**	**CRS400359**	**CRS400393**
**Structure**	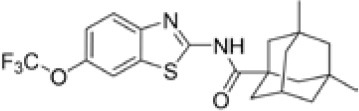	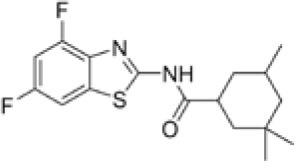	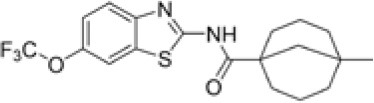	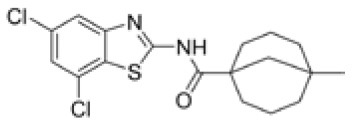
**RAPID-GROWING NTM, MIC (**μ**g/mL)**				
*M. abscessus* ATCC 19977	0.25	0.5	≤ 0.06	0.03
*M. abscessus massiliense* 119	0.25	0.5	≤ 0.06	0.03
*M. chelonae* 93	0.5	0.25	0.12	0.03
*M. fortuitum* 41	0.12	0.25	0.12	0.06
*M. peregrinum* ATCC 700686	≤ 0.06	0.12	0.12	0.03
**SLOW-GROWING NTM, MIC (**μ**g/mL)**				
*M. avium* 101	2	16	2	2
*M. intracellulare* 1956	1	8	2	2
*M. chimaera* 1502055	1	NT	2	1
*M. tuberculosis* H37Rv mc^2^ 6206	0.5	1	0.5	≤ 0.12
**SPECTRUM Of ACTIVITY, MIC (**μ**g/mL)**				
*S. aureus* ATCC 29213	>64	>64	>64	>64
*E. faecalis* ATCC 29212	>64	>64	>64	>64
*S. pyogenes* ATCC 19615	>64	>64	>64	>64
*S. pneumoniae* ATCC 49619	64	8	32	32
*E. coli tolC* CGSC 5633^a^	>64	>64	>64	64
*E. coli* ATCC 25922 + PMBN^b^	>64	>64	>64	>64
*P. aeruginosa* ATCC 35151	>64	>64	64	64
*C. albicans* ATCC 10231	NT	NT	64	>64
Anaerobes (*n* > 20 strains)^c^	>16	NT	NT	>32
**ADMET PROPERTIES**				
HepG2 cytotox., IC_50_ (μM [μg/mL])	69 (29)	NT	10 (4)	>10 (>4)
Hemolysis (μg/mL)	128	128	>128	128
Solubility (μM), phosphate buffer	<3	3	<3	<3
Protein (albumin) binding (%)	>95	>95	>95	>95
Protein (serum) binding (%)	>95	>95	>95	>95
Metabolic stability (μL/min/mg)^d^	124	NT	NT	NT
CYP2B6 inhibition, 10 μM (%)	<50	NT	30	39
CYP3A inhibition, 10 μM (%)	<50	NT	0	26
**OTHER PROPERTIES**				
Molecular weight (M.W.)	424.5	338.42	398.4	383.3
cLogP	6.67	5.31	6.70	6.48

a *Efflux mutant strain of E. coli*.

b *Permeabilized strain of E. coli; PMBN, polymyxin B nonapeptide*.

c *Including genus Clostridium, Bifidobacterium, Fusobacterium, Peptostreptococcus, Bacteroides, Lactobacillus, Veillonella, Blautia, Parvimonas, Finegoldia, Coprococcus, Actinomyces, Eubacterium, and Prevotella*.

d *Clearance measured in human liver microsome assay*.

*NT, not tested*.

**Table 2:** *In vitro* Activity (MIC, μg/mL) of benzothiazole amides and control agents against rapid-growing NTM clinical Isolates.

**Compound**	***M. abscessus*** **(*****n*** = **20)**		***M. chelonae*** **(*****n*** = **11)**		***M. fortuitum*** **(*****n*** = **11)**		**Other NTM (*n* = 12)^a^**	
	**MIC range**	**MIC_90_^b^**	**MIC range**	**MIC_90_**	**MIC range**	**MIC_90_**	**MIC range**	**MIC_90_**
CRS400226	0.25–2	0.5	0.5–0.5	0.5	0.12–0.5	0.5	0.12–1	0.5
CRS400153	0.5–2	1	0.5–1	1	0.5–0.5	0.5	0.25–1	1
CRS400359	0.06–2	0.25	0.25–0.5	0.25	0.06–1	1	0.12–2	0.5
CRS400393	≤ 0.03– 0.25	0.25	0.12–0.12	0.12	≤ 0.03–0.5	0.25	0.12–0.5	0.5
Amikacin	1–>16	16	16–>16	>16	0.5–4	2	0.5–16	16
Linezolid	0.5–>8	>8	1–8	8	0.5–>8	>8	0.5–>8	>8
Azithromycin	0.12–>8	>8	0.12–0.5	0.5	0.12–>8	>8	0.12–>8	>8

a *Other rapid-growing NTM included two strains each of M. peregrinum, M. mucogenicum, M. massiliense, M. bolletii, M. phocaicum, and M. porcinum*.

b *MIC_90_ defined as the minimum drug concentration that inhibited growth of ≥90% of the strains tested*.

### Mode of action and target identification

**Figure 1:**
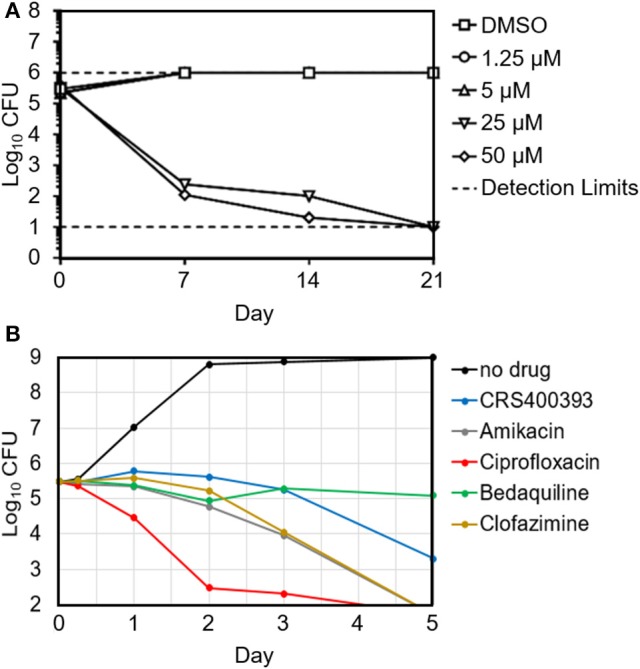
Time-kill kinetics of *Mtb* H37Rv in the presence of increasing concentrations of CRS400226 **(A)** and of *Mabs* 21 in the presence of CRS400393 or other antimycobacterial agents at 10x MIC **(B)**, indicating bactericidal activity.

Benzothiazole amides showed bactericidal activity in time-kill assays. CRS400226 at concentrations of 25 μM (10 μg/mL) caused a reduction in *Mtb* of 3 Log_10_ CFU within 7 days. CRS400393 was bacteriostatic against *Mabs*, for 3 days, followed by a drop of 2 Log_10_ CFU after 5 days. Importantly, no regrowth was observed following a single addition of compound due to emergence of resistance (Figure 1). *In vitro* checkerboard synergy assays of the benzothiazole amides showed additive effects with many commonly used antimycobacterial agents (amikacin, ciprofloxacin, azithromycin, tobramycin, clofazimine, linezolid, and cefoxitin), and indifference when combined with doxycycline or bedaquiline (Supplementary Table S3). Mode-of-action studies were performed with CRS400153 and CRS400226, which represent the cyclohexyl and the adamantyl subseries of benzothiazole amides, respectively. Evidence was obtained that compounds from both subseries at 2x MIC and 10x MIC affect the transfer of mycolic acids to their cell envelope acceptors in both *Mabs* and *Mtb*, most likely through the inhibition of the trehalose monomycolate transporter, MmpL3. Indeed, metabolic labeling with [^14^C]acetate of *Mabs* ATCC19977 cultures either untreated or treated with benzothiazole amides at 2x and 10x MIC resulted in a concentration-dependent inhibition of mycolic acid transfer to both arabinogalactan and trehalose dimycolates, a hallmark of MmpL3 inhibitors (Supplementary Figure S4). Moreover, spontaneous *Mabs* ATCC19977 mutants resistant to CRS400153 were isolated with a frequency of 6 × 10^−9^ at 4x MIC and were found to harbor non-synonymous mutations in MmpL3 (L551S, I306S, or A309P). These mutations were sufficient to confer reduced susceptibility (4 to >32-fold MIC increase) to CRS400153 and CRS400359 when introduced via recombineering into the isogenic background of Mabs ATCC19977. Moreover, testing of 15 additional analogs from the same compound subclass indicated that all of them displayed 8–64-fold reduced activity against CRS400153-resistant mutants harboring mutations in MmpL3. Interestingly, the same A309P and L551S mutations were previously reported to decrease 16- to 64-fold the susceptibility of *M. abscessus* to MmpL3 inhibitors of the indole-2-carboxamide and piperidinol series (Dupont et al., 2016; Franz et al., 2017) (unpublished data). The I306 residue has not previously been associated with resistance to MmpL3 inhibitors but maps in the fifth transmembrane region of the transporter, very close to A309 and to a conserved serine residue (S302 in *M. abscessus*, S288 in Mtb) shown to confer resistance to a variety of MmpL3 inhibitors in Mtb (Belardinelli et al., 2016). These data strongly suggest that compounds from the structural class of benzothiazole cyclohexyl amides such as CRS400153 kill *M. abscessus* through the direct or indirect inhibition of MmpL3. The fact that CRS400153 showed no effect on the membrane potential and electrochemical pH gradient of Mtb intact cells and inverted membrane vesicles (data not shown) supports a direct mechanism of inhibition of the transporter (Li et al., 2018).

### *In vivo* efficacy and tolerability

**Figure 2:**
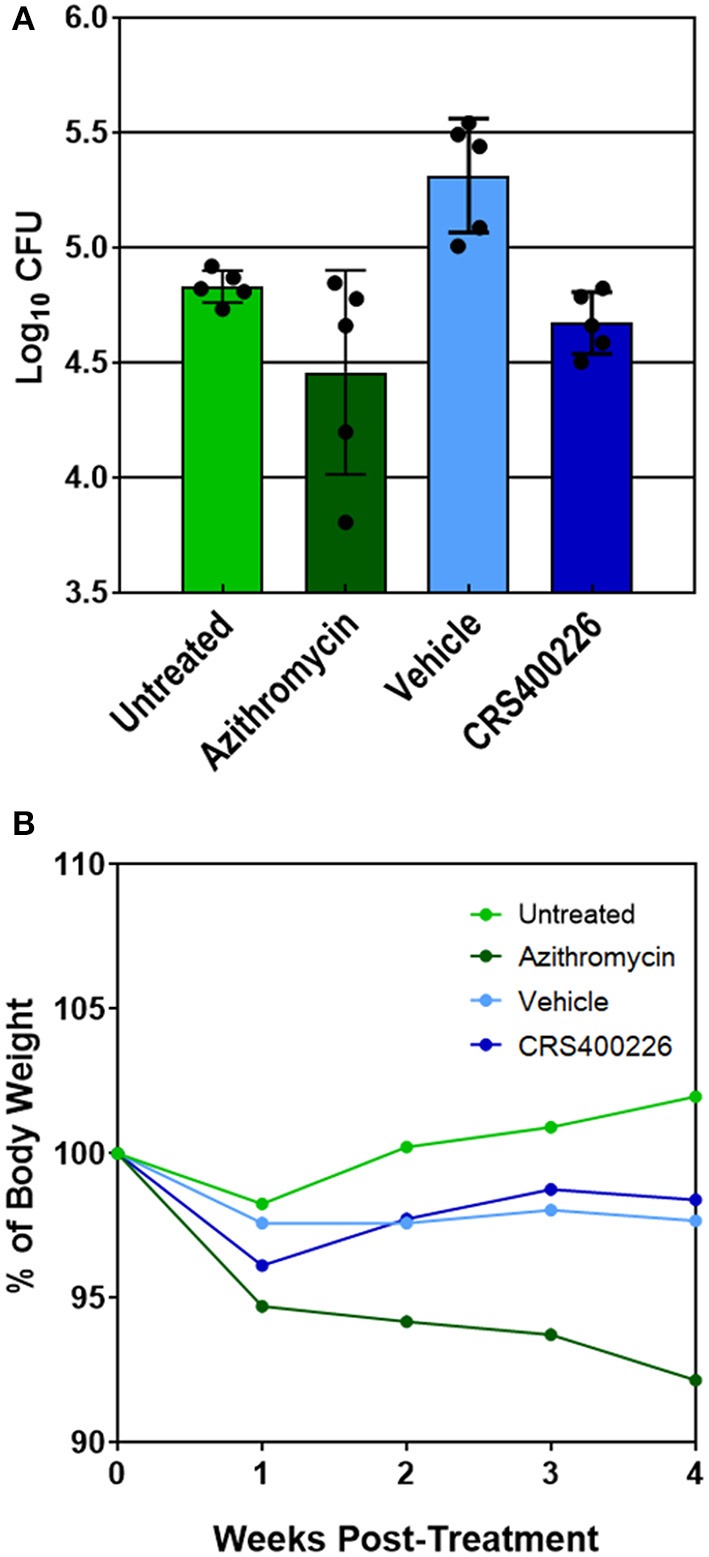
GM-CSF KO mouse model of *Mabs* lung infection demonstrating efficacy **(A)** and tolerability **(B)** of CRS400226. The Penn Century microsprayer was used for delivery of 5 × 10^5^ to 1 × 10^6^ bacteria to the lungs. Starting 10 days later, the test compound was dosed once daily over 4 weeks by intratracheal microspray administration as 5% corn oil/saline emulsion. CRS400226 at 25 mg/kg daily for 28 days resulted in a statistically significant (*p* = 0.0005) reduction of 0.64 Log_10_ CFU compared to the vehicle control.

Several compounds were screened in mouse PK and tolerability studies to evaluate various doses and routes of administration. Although essentially insoluble in water, we found CRS400226 was soluble in corn oil at concentrations up to 250 mg/mL. Oral dosing of this compound at 100 mg/kg in female CD-1 mice resulted in very low plasma levels of 2.3 μg/mL at 1 h and 2.0 μg/mL at 8 h. Although highly tolerated, it became clear that the lipophilic nature and high protein binding of the compounds precluded oral routes of administration for efficacy. Instead, intrapulmonary delivery methods for treatment of lung infections such as NTM and *Mtb* were explored. CRS400226 was synthesized in multi-gram quantities to support *in vivo* studies and was further developed for this delivery pathway. These initial attempts to formulate CRS400226 as a corn oil/saline emulsion afforded the opportunity to obtain proof-of-concept *in vivo* efficacy. We assessed CRS400226 against *Mabs* 21 in a 28-day mouse model of chronic NTM lung infection in granulocyte macrophage colony stimulated factor-knockout (GM-CSF KO) mice, which represent a clinically relevant model of human infection (De Groote et al., 2014b). Actual dose deposited in the lungs was 4.53 Log_10_ CFU, which increased to 4.98 Log_10_ CFU after 10 days of infection, as determined in groups of three mice. Intratracheal administration of CRS400226 at 25 mg/kg once daily, 5 days per week for 4 weeks resulted in a statistically significant (*p* = 0.0005) reduction of 0.64 Log_10_ CFU compared to the vehicle control (Figure 2A). Azithromycin at 100 mg/kg achieved 0.38 Log_10_ CFU reduction when compared to the untreated group (*p* = 0.05), though the data points in the azithromycin group were more scattered than in the other groups. There was no statistical difference (*p* = 0.17) in the bacterial burden between the CRS400226 and azithromycin treated groups. No animals died during the 4-week course of the efficacy model, though all treatment groups demonstrated a slight decrease in weight ranging from 1.6 to 7.8%. CRS400226 was well tolerated up to 4 weeks of treatment, without any observed adverse side effects (Figure 2B). Histology showed that the azithromycin treated animals had only minimal pathologic evidence of inflammatory infiltrates with one animal displaying normal appearing lung tissue. CRS400226 treated animals had only very mild areas of airway-centric inflammation compared to the peribronchial inflammatory infiltrate seen in the vehicle treated group. The untreated and vehicle treated lung tissue demonstrated scattered nodules and more significant inflammatory infiltrates. In many of the animal lungs, evidence of bronchiectasis was observed and inflamed peri-bronchial lymphoid tissue (Supplementary Figure S5).

## Discussion

We have developed a lead series that exhibits broad antimycobacterial activity, which could revolutionize the treatment of NTM, while simultaneously adding much-needed novel agents to the *Mtb* treatment armamentaria. The strength of the benzothiazole amide series is that advanced lead compounds are very potent *in vitro* and mid-stage compounds showed *in vivo* efficacy. Good potency of the benzothiazole amides was demonstrated against a collection of rapid growing NTM clinical isolates which included many strains that were resistant to amikacin, linezolid, or azithromycin illustrating the high prevalence of non-susceptibility of NTM to many antibiotics commonly used to treat NTM infections and the need for novel agents whose activity is not affected by existing resistance mechanisms. Apparent MIC values against slow-growing NTM and *Mtb* were 3higher compared to the MIC values seen against rapid-growing NTM. This has been attributed to differences in the media used to test the different mycobacterial species, specifically to the presence of albumin (5 mg/mL) in the OADC supplement used for slow- growing NTMs and for *Mtb*, causing an MIC shift of the relatively highly (>95%) protein-bound compounds (Supplementary Table S6). CRS400393 showed additivity and no antagonism when combined with other antimycobacterial agents, an essential property for treating chronic NTM infections that often require combination drug regimens. Another favorable property of the benzothiazole amides is their narrow spectrum, since such a mycobacterial-specific agent may prove more tolerable for prolonged duration of therapy, i.e., by not affecting the beneficial gut microbiome that protects the host from gastroenteric side effects such as *Clostridium difficile* infections that often arise from exposure to broad-spectrum antibiotics.

The apparent target of benzothiazole amides, MmpL3, is a mycolate transporter essential for assembly of the outer membrane and has high potential to improve and shorten current drug-susceptible and drug-resistant tuberculosis chemotherapies (Li et al., 2016, 2018). Several MmpL3 inhibitors with different chemical scaffolds have been reported (Poce et al., 2013, 2016; Li et al., 2014; Rayasam, 2014; Stec et al., 2016). One compound, SQ109, which is unrelated to our compound series, has been in clinical development through Phase II (Sacksteder et al., 2012; Tahlan et al., 2012). The nature of the membrane-bound MmpL3 target favors lipophilic and therefore poorly water-soluble inhibitors. Efforts to optimize oral drug-like properties such as improving compound solubility have largely resulted in loss of activity. This potential limitation had been discovered through detailed SAR of the early lead compound CRS400153, by exploring hydrophilic substitutions at the 4-, 5-, 6-, and 7-position of the benzothiazole moiety, such as a hydroxyl, tertiary amine, and ether or ester groups. In all cases, these hydrophilic substitutions resulted in MIC > 64 μg/mL against *M. abscessus* compared to MIC = 0.5 μg/mL for the 4,6-diF-benzothiazole analog CRS400153, except for the 7-OH benzothiazole analog which retained some activity with an MIC range of 2–16 μg/mL against *M. abscessus* (compound #37) (Graham et al., 2018). Our lead compounds are very potent, but have very low solubility (≤3 μM), high protein binding (MIC ≥ 16 μg/mL in 50% serum) and low oral bioavailability due to their lipophilic nature, precluding their utility as oral agents. Therefore, further development will focus on inhalational therapy for NTM first and possibly development of a topical formulation later. Similar approaches have been reported for aerosolized liposomal amikacin (Rose et al., 2014) and for inhaled dry powder capreomycin with the potential of rendering patients non-infectious (Dharmadhikari et al., 2013). Our proof-of-concept efficacy study used corn oil for formulation, and we noted an increased bacterial burden in the vehicle treated group compared to the untreated group, which is likely attributable to corn oil being a carbon source for mycobacteria, increasing their replication; hence, developing a more sophisticated formulation will be paramount.

The desired target product profile for this drug includes (1) broad-spectrum coverage of the clinically-relevant mycobacteria *Mabs*, MAC and *Mtb*, (2) bactericidal activity, (3) antibacterial potency equal or better compared to antibiotics currently used in the standard of care for NTM infections, (4) suitability for inhaled therapy via aerosol, (5) dosing frequency preferably once or twice a day, (6) excellent safety profile suitable for treating chronic infections and for pediatric use, (7) low propensity for resistance development, (8) compatibility with other antibacterial drug cocktails, and (9) shelf-life of at least 2 years. Our data indicate that the benzothiazole amides already meet the criteria regarding the *in vitro* antimyobacterial activity and low resistance frequencies. The next steps will be the evaluation of the compounds regarding efficacy, safety, tolerability, and chemical stability. Cytotoxicity studies, however, have been limited by the low solubility of the compounds, which made testing at high concentrations impossible, and potential effect may be masked due to the high protein binding of the compounds.

In conclusion, the limitations of the scaffold are high lipophilicity, high protein binding, and poor PK with oral dosing. Such challenges in drug development can often be overcome by exquisite potency of the agents and improved formulation and delivery methods. A recent example of an antimycobacterial drug with some undesirable physicochemical properties is bedaquiline, which nonetheless was developed successfully and became the first FDA-approved TB drug in 40 years (Palomino and Martin, 2013).

*In vivo* efficacy and tolerability of mid-stage lead compound CRS400226 provides exciting evidence of the potential for this series; new analogs have already shown further potency increases, providing a solid foundation for further optimization. Next steps will include testing of pharmacokinetic properties, metabolic stability, toxicity and efficacy of the most recent potent compounds such as CRS400393 against NTM and *Mtb* as inhaled therapy, hopefully leading to IND-enabling studies.

## Author contributions

MD, UO, XS, JD, and TJ conceived the project and designed the strategy. Experiments were carried out by CW, JG, JD, and XS (chemical synthesis and SAR), TH, CY, and UO (*in vitro* susceptibility and other microbiological assays), WL and MJ (mode-of-action studies) and MG-J (*in vivo* efficacy). WR was Project Manager and assisted with data analysis. MD and UO wrote the manuscript.

### Conflict of interest statement

UO, XS, JD, TJ, CW, JG, JD, XS, TH, and CY are employees of Crestone, Inc. The remaining authors declare that the research was conducted in the absence of any commercial or financial relationships that could be construed as a potential conflict of interest.
