# Erratum: Optimization and Lead Selection of Benzothiazole Amide Analogs Toward a Novel Antimycobacterial Agent

**DOI:** 10.3389/fmicb.2019.02298

**Published:** 2019-10-04

**Authors:** Frontiers Production Office

**Affiliations:** Frontiers Media SA, Lausanne, Switzerland

**Keywords:** NTM, tuberculosis, antibacterial therapeutics, benzothiazole amide, MmpL3, efficacy, tolerability, aerosol

Due to a typesetting error, the author order was changed during the production of the article. The correct order is: Mary A. De Groote, Thale C. Jarvis, Christina Wong, James Graham, Teresa Hoang, Casey L. Young, Wendy Ribble, Joshua Day, Wei Li, Mary Jackson, Mercedes Gonzalez-Juarrero, Xicheng Sun and Urs A. Ochsner.

The publisher apologizes for this error. The original article has been updated.

