# Supplementary material for: Optimization and Lead Selection of Benzothiazole Amide Analogs Toward a Novel Antimycobacterial Agent
Source: Front Microbiol. 2018 Sep 20;9:2231. doi: 10.3389/fmicb.2018.02231 (PMC6158578; doi:10.3389/fmicb.2018.02231)
Supplement: Supplementary file 1 [file Presentation_1.PDF]

Supplementary Figure S1. MIC distributions for benzothiazole amides and control antibiotics against a collection of n=54 rapid-growing NTM clinical isolates, including n=20 *M. abscessus*, n=11 *M. chelonae*, n=11 *M. fortuitum*, and n=12 other rapid-growing NTM. The benzothiazole amides demonstrated a tight MIC range (green), while a bimodal MIC distribution due to non-susceptible strains was observed for amikacin, linezolid and azithromycin.

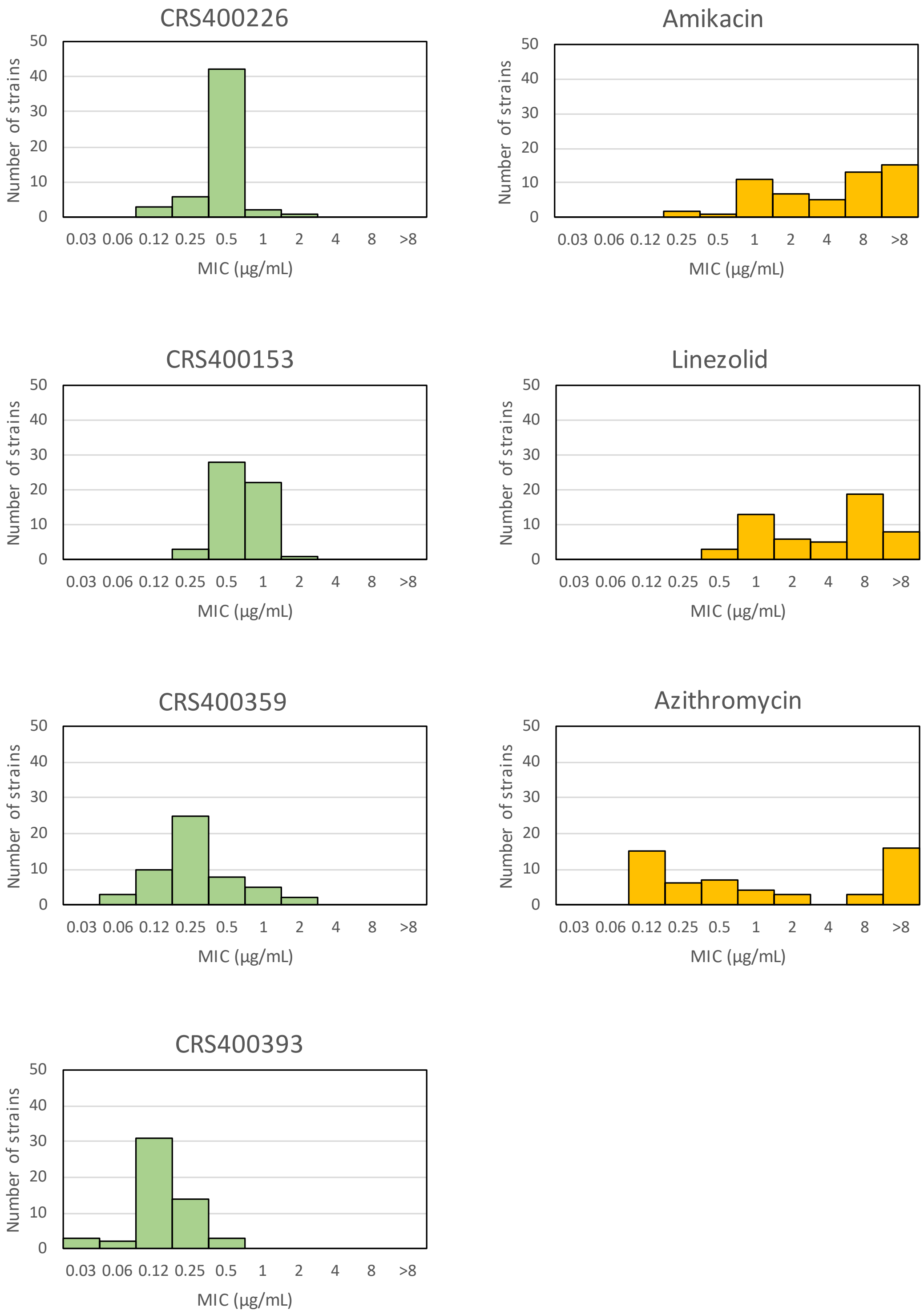

Supplementary Table S2. *In vitro* activity of CRS400226 under different testing conditions and against drug-resistant strains of *Mtb*. IC_{90} values, defined as drug concentrations that inhibited 90% of growth compared to no-drug controls were determined from a dose-response growth inhibition assay in the presence of increasing drug concentrations.

**Condition/strain**	**IC_{90} (µg/mL)**	**MBC (µg/mL)**
Aerobic	4.0	10.6
Low oxygen (Wayne hypoxic model)	6.8	NT
Intracellular *Mtb*	0.8	NT
Fluoroquinolone-resistant *Mtb*	3.7	NT
Isoniazid-resistant *Mtb* strain 1	3.9	NT
Isoniazid-resistant *Mtb* strain 2	4.2	NT
Rifampin-resistant *Mtb* strain 1	3.9	NT
Rifampin-resistant *Mtb* strain 2	4.7	NT

Supplementary Table S3. *In vitro* ‘checkerboard’ synergy testing of CRS400393 with other antibiotics.

Antibiotic	FIC Index	Interpretation
Amikacin	1	additive
Ciprofloxacin	0.56	additive
Azithromycin	1	additive
Doxycycline	2	indifferent
Sulfamethoxazole	0.56	additive
Tobramycin	0.75	additive
Bedaquiline	2	indifferent
Clofazimine	0.52	additive
Linezolid	0.56	additive
Cefoxitin	0.56	additive

Supplementary Figure S4. Metabolic labeling with [^{14}C]acetate of *Mabs* ATCC19977 cultures either untreated or treated with benzothiazole amides at 2x and 10x MIC. A. Total lipids; TMM, trehalose mono-mycolate; TDM, trehalose di-mycolate. B. Cell wall-bound α and α’ mycolates.

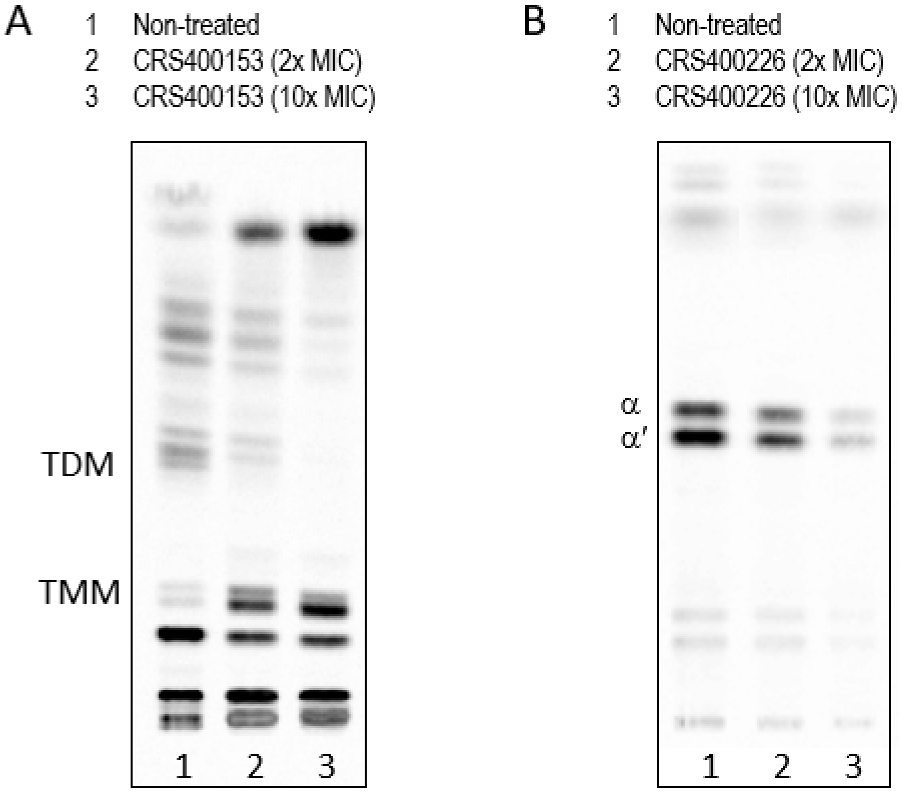

Supplementary Figure S5. Histopathology of lungs (middle right lobes) of GM-CSF knockout mice infected with *M. abscessus* by the intrapulmonary route and treated for 4 weeks (5 days/week) with the respective agents. Azithromycin was administered by oral gavage. CRS400226 and vehicle were delivered via intrapulmonary microspray administration. At the time of necropsy, the right middle lung lobes from 5 animals per group were placed in 4% paraformaldehyde, followed by paraffin embedding and hematoxylin and eosin staining. Image was taken at 4x.

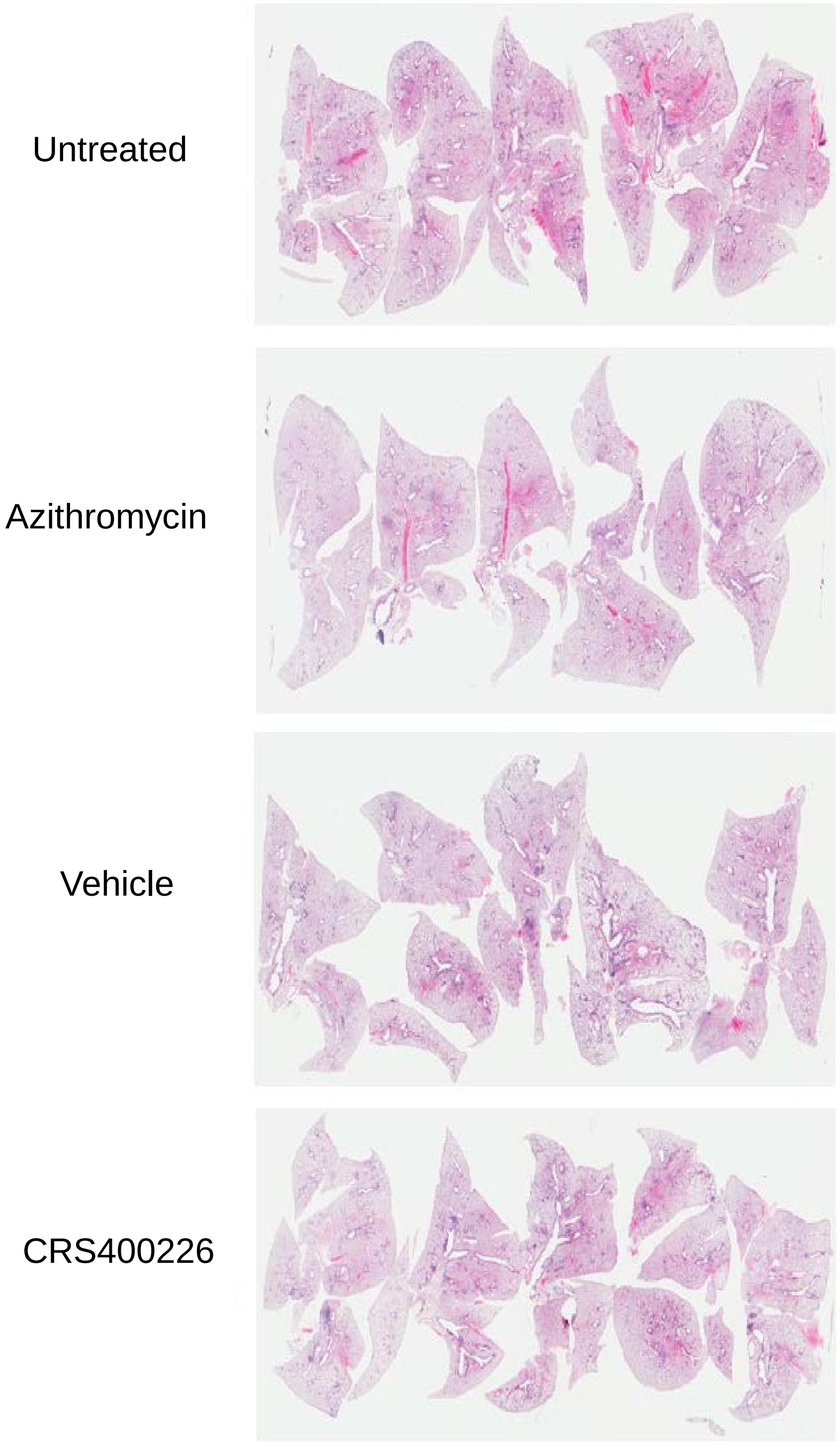

Supplementary Table S6. MIC values for benzothiazole amides and control agents against *M. abscessus*. MIC shifts of the highly protein-bound benzothiazole amides were observed in Middlebrook medium (7H9+OADC) compared to Muller Hinton broth (MHB) due to the presence of 5 mg/mL albumin in the OADC supplement.

**Compound**	***M. abscessus* 1**		***M. abscessus* 21**		***M. abscessus* 79**	
	**MHB**	**7H9 + OADC**	**MHB**	**7H9 + OADC**	**MHB**	**7H9 + OADC**
CRS400226	0.25	8	0.25	8	0.25	2
CRS400359	0.06	1	0.06	2	0.06	0.25
CRS400393	0.06	1	0.03	1	0.03	0.5
Amikacin	8	16	2	4	2	4
Ciprofloxacin	4	8	4	4	NT	2
